# Case Report: Chemotherapy-Associated Systemic Sclerosis: Is DNA Damage to Blame?

**DOI:** 10.3389/fmed.2022.855740

**Published:** 2022-02-24

**Authors:** Amy X. Du, Robert Gniadecki, Jan Storek, Mohamed Osman

**Affiliations:** ^1^Faculty of Medicine and Dentistry, University of Alberta, Edmonton, AB, Canada; ^2^Division of Dermatology, Department of Medicine, University of Alberta, Edmonton, AB, Canada; ^3^Division of Hematology, Department of Medicine, University of Calgary, Calgary, AB, Canada; ^4^Division of Rheumatology, Department of Medicine, University of Alberta, Edmonton, AB, Canada

**Keywords:** scleroderma, systemic sclerosis, chemotherapy, DNA damage, hematopoietic cell transplantation

## Abstract

Systemic sclerosis, also known as scleroderma, is an autoimmune disease characterized by cutaneous and visceral fibrosis, immune dysregulation, and vasculopathy. Generally, the degree of skin fibrosis is associated with an increased likelihood of visceral organ involvement. Its pathogenesis is poorly understood; however, it is clear that changes in both the innate and adaptive immune responses are associated with fibroblast dysfunction and vascular damage. Further, DNA damage has been postulated as one of the triggering factors in systemic sclerosis, although the association of DNA damage with the progression of this disease is more poorly established. Recently, abnormal DNA damage response repair pathways have also been identified in patients with systemic sclerosis, suggesting that cells from patients with this disease may be more susceptible to DNA damaging agents. Chemotherapeutic drugs and other DNA damaging agents have been associated with the development of systemic sclerosis, as these agents may provide additional “hits” that promote abnormal DNA damage responses and subsequent inflammatory changes. Herein, we present the case of a 39-year-old female who developed scleroderma after the treatment of her breast cancer with chemotherapeutic agents. Her scleroderma was subsequently successfully treated with autologous hematopoietic stem cell transplantation. We also completed a literature review for previously published cases of chemotherapy associated with systemic sclerosis and highlighted a role of DNA damage in promoting the disease. Our case is the first case of chemotherapy associated with systemic sclerosis treated with hematopoietic stem cell transplantation.

## Introduction

Systemic sclerosis (SSc), also known as scleroderma, is an autoimmune disease characterized by cutaneous and visceral fibrosis, immune dysregulation, and vasculopathy (1). Early in the disease (<3 years from its onset), patients may develop skin fibrosis that is not extensive, although some patients may also develop rapidly progressive skin fibrosis [or early diffuse SSc (edSSc) (2–4)], which is characterized by skin thickening extending beyond the elbows, and often the trunk over a short disease duration. Generally, the degree of skin fibrosis is associated with an increased likelihood of visceral organ involvement and mortality (5).

**Figure 1:**
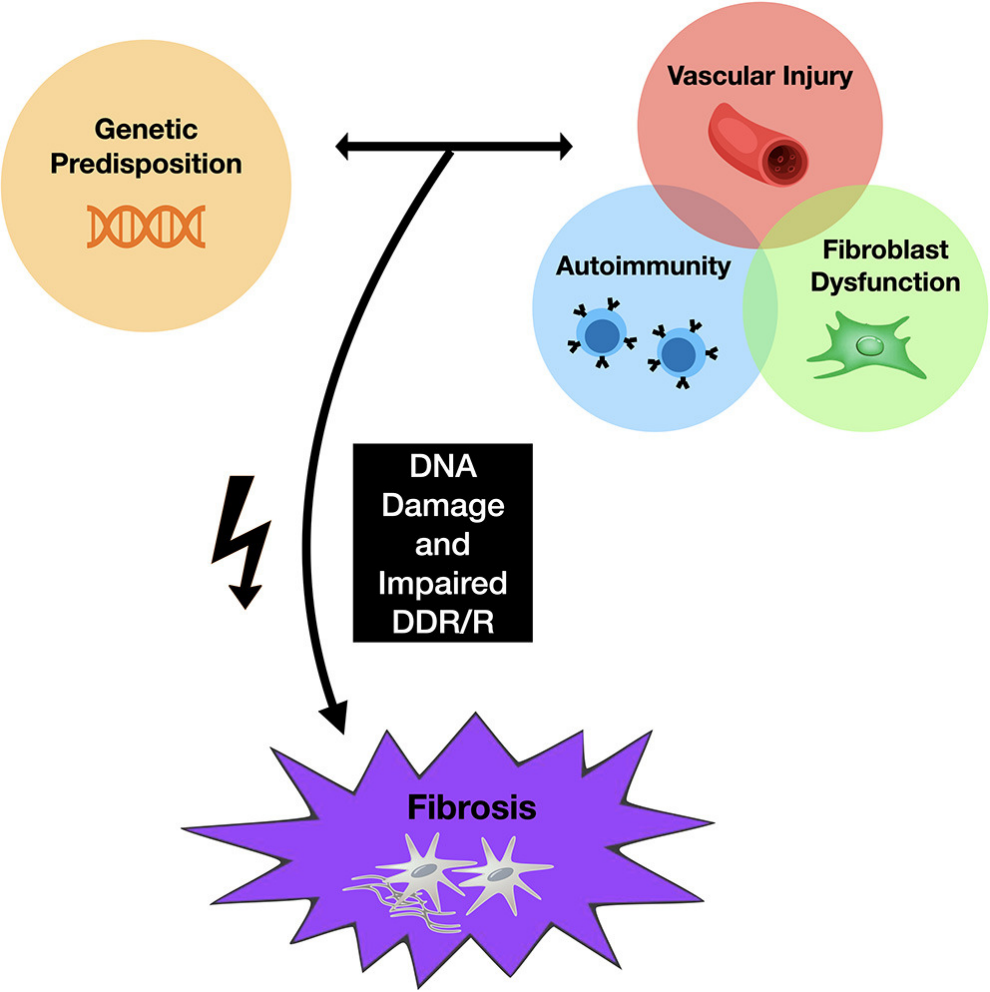
Proposed mechanism of skin fibrosis associated with chemotherapy. Genetic predisposition to SSc, along with changes in both the innate and adaptive immune responses, promote fibroblast dysfunction and vascular damage, leading to fibrosis. Abnormal DNA damage response repair pathways have also been identified in patients with SSc, suggesting that cells from patients with SSc may be more susceptible to DNA damaging agents. Chemotherapeutic agents may provide the additional cellular “hit” that promotes abnormal DNA damage responses and subsequent inflammatory changes.

The pathogenesis of SSc is poorly understood, although it is clear that changes in both the innate and adaptive immune responses are associated with fibroblast dysfunction and vascular damage (6). DNA damage, promoted by reactive oxygen species (ROS), has also been postulated as one of the triggering factors in SSc (7–10), although, the association of DNA damage with the progression of SSc is poorly established. Recently, abnormal DNA damage response repair (DDR/R) pathways have been identified in patients with SSc, suggesting that cells from patients with SSc may be more susceptible to DNA damaging agents (11, 12) (Figure 1).

Chemotherapy functions to avoid malignant invasion and metastasis by inhibiting cell proliferation and tumor growth using traditional agents aimed at inhibiting DNA, RNA, or protein synthesis (13). This process is what leads to their cytotoxic effects and subsequent adverse reactions. Chemotherapeutic drugs, including, but not limited to, alkylating agents, antimetabolites, mitotic inhibitors, and anthracyclines, have been associated with the development of scleroderma, with the taxane group of medications, in particular, being highly associated with this disease (14–16). Thus, these agents may provide the additional cellular “hit” that promotes abnormal DNA damage responses and downstream inflammatory signals that promote characteristic fibroblast and immune cell abnormalities described in SSc (Figure 1) (1).

Here, we present a case of chemotherapy-associated scleroderma that was subsequently successfully treated with autologous hematopoietic stem cell transplantation (HSCT). As part of our description, we have completed a brief review of the literature for previously published cases of chemotherapy associated with the development of skin fibrosis, and we describe the role of DNA damage in the pathogenesis of SSc. To the best of our knowledge, this is the first case of edSSc associated with chemotherapy and this is the first demonstration of subsequent treatment using HSCT.

## Case Report

A 39-year-old Caucasian female was diagnosed with biopsy-proven grade III invasive ductal carcinoma cancer of the right breast, and subsequently underwent a right mastectomy. Then, she was treated with three cycles of 5-fluorouracil, epirubicin, cyclophosphamide, and docetaxel chemotherapy. She received 45 Gy of radiation therapy to the affected areas, which was complicated by mild lymphedema.

During her last two cycles of chemotherapy, the patient complained of swollen or “puffy” fingers bilaterally, resembling dactylitis, leaving her unable to fully extend her fingers. This was associated with bilateral leg and foot swelling. One month later, she presented with symptoms of numbness and poor perfusion in the areas distal to the metacarpophalangeal joints on both hands that appeared to have a biphasic nature (ischemic and erythema phase) that was highly suggestive of Raynaud's phenomenon. There was no cyanotic phase affecting her fingers at the time. The patient's symptoms were most notably precipitated in the shower and by cold temperatures. Additionally, tightness of her mouth, neck and face were noted. She was unable to abduct her arms above her head. Furthermore, she developed gastroesophageal reflux not associated with symptoms of dysphagia or looser, more frequent bowel movements. Notably, the patient developed progressive skin fibrosis (starting from her hands and moving to her trunk) associated with profound skin itchiness which led to impairment and difficulties with her activities of daily living.

**Figure 2:**
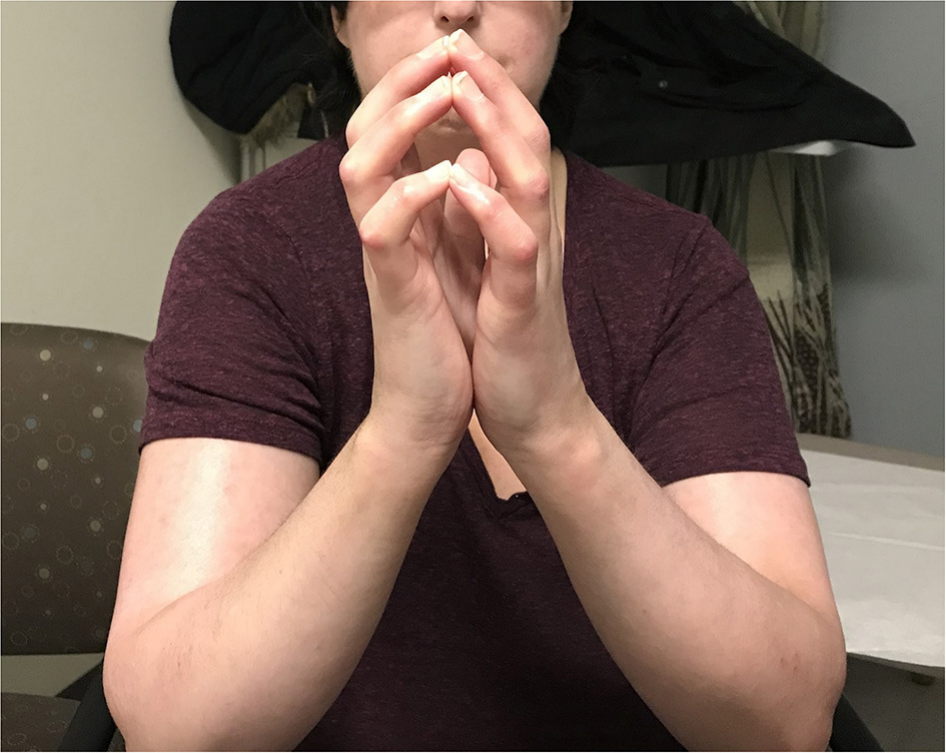
On exam, the patient had no digital ulcers, but her hands were fixed in flexion, with sclerodactyly and a positive “prayer sign”. There is also evidence of skin tightness in the bilateral upper extremities extending to the elbows.

**Figure 3:**
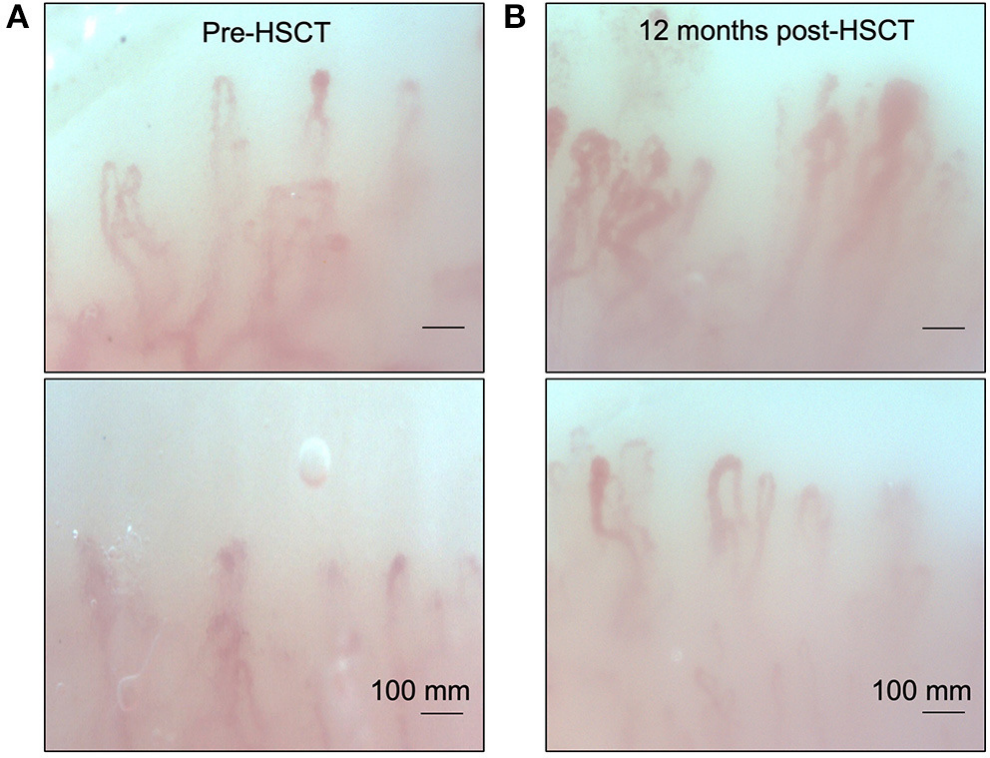
Nailfold video capillaroscopy for our patient showing a late pattern described in SSc. **(A)** Note the capillary disorganization and decreased capillary density present before autologous hematopoietic stem cell transplantation. **(B)** Repeat nailfold video capillaroscopy examination 12 months post HSCT. Note the increased capillary density and improved organization after transplant.

Her clinical examination revealed skin tightness in the bilateral upper extremities extending to the elbows. Some patches of skin were associated with calcinosis cutis. She had no digital ulcers, but her hands had evidence of sclerodactyly with reduced extension compatible with a positive prayer sign (Figure 2). Investigations were in keeping with SSc, as suggested by anti-RNA polymerase III antibodies [RP11 and RP155, performed by a reference laboratory Mitogen Laboratories (MitogenDx, Calgary, AB, Canada)]. Other SSc-specific autoantibodies (e.g., anti-Scl70, anti-fibrillarin, anti-Th/T0, and anti-centromere) were absent. Echocardiogram, CT of the chest, abdomen and pelvis were also normal (specifically there was no evidence of breast cancer recurrence) except for mild lung fibrosis only in the breast radiation field and severe hepatic steatosis. Forced vital capacity (FVC) was 63%, likely due to chest wall fibrosis. Gastroscopy did not reveal the presence of esophagitis or strictures. Esophageal manometry revealed hypomotility (40% swallows failed and 20% swallows were weak). Nailfold video capillaroscopy showed decreased capillaries in most digits [mean capillary density 4.2 capillaries per mm, Figure 3A—pattern described as a “late capillary SSc pattern” (17)]. Her pre-HSCT modified Rodnan skin score (mRSS) was 31/51.

She was started on mycophenolate mofetil (MMF) 1,000 mg PO b.i.d. for immunosuppression, with good drug tolerance. Antihistamines and low-dose prednisone, at 5 mg PO daily, were also initiated as the patient's pruritus was significantly affecting her quality of life. After 14 months from symptom onset, she was referred for evaluation of autologous HSCT therapy. It was felt that although her presentation was atypical, her likelihood of survival with conventional therapy was reduced compared to HSCT with an estimated 5-year survival of 85% with stem cell transplant vs. 50–75% with conventional immunosuppressive therapy. Her quality of life was expected to be superior after stem cell transplant. The patient underwent autologous hematopoietic cell transplantation (HSCT) (18) ~18 months after her initial symptoms of skin thickening. Her course was complicated by a catheter-induced left internal jugular vein thrombosis associated with heparin-induced thrombocytopenia and thrombosis (HITT). She was started on fondaparinux for this complication. By 8 days post-transplant, she had become neutropenic but was initiated on granulocyte colony stimulating factor (G-CSF) and subsequently recovered her cell counts with no further complication. After about 6 months post-transplant, the patient still endorsed some shortness of breath on exertion but overall was feeling less fatigued. FVC at 1 year post-transplant was 67% predicted. She noticed some improvement in her skin tightening but still had flexion contractures at several joints. She also described ongoing Raynaud's symptoms, but minimal digital ulcerations. She had ongoing gastroesophageal reflux that had not improved post-transplant. At ~18 months post-transplant, her mRSS has markedly improved (15/51). Post-transplant nailfold capillaroscopy showed improvement with capillary density at 5.8 capillaries/mm with mild apical enlargement (~32 microns in 30% of capillaries), minimal giant capillaries and microhemorrhages (Figure 3B). She continues to be followed as an outpatient and continues to exhibit subjective clinical improvement.

## Discussion

Chemotherapy-associated skin fibrosis has been previously described in the literature, with cutaneous fibrosis being one of the most common symptoms, and taxane chemotherapeutic agents being the primary offender (5, 16, 19–23). The earliest cases describing chemotherapy associated skin fibrosis were published by Battafarano et al. in 1995, describing three patients who developed diffuse lower extremity edema and subsequent scleroderma-like changes after receiving multiple cycles of docetaxel therapy for various malignancies (16). Rheumatoid factor, antinuclear antibodies, anticentromere, and topoisomerase antibodies were not present in any patient, and the discontinuation of docetaxel correlated with resolution of edema and softening of the skin.

The occurrence of edSSc associated with chemotherapeutic agents manifesting as severe skin fibrosis, the presence of specific autoantibodies, and vasculopathy is rare. Indeed, to the best of our knowledge, our case is the first case of edSSc (3, 4) in this setting. Case reports of SSc or scleroderma-like changes occurring after treatment with various other chemotherapeutic drugs, such as bleomycin (24–26), gemcitabine (27–29), and pemetrexed (30–32), have also been published, however, none of these cases had associated vasculopathy and SSc-specific autoantibodies. Moreover, our case was successfully treated with HSCT, which further underpins the utility of HSCT in the management of rapidly progressive SSc.

The mechanisms by which various chemotherapeutic agents induce specific scleroderma-like skin changes remain unclear. However, a driver associated with skin and visceral organ fibrosis may be DNA damage (4, 11, 33, 34). DNA damage signals are associated with dysregulated type I interferon activation (35) and downstream interleukin 6 (IL-6) release (36), which are known to be associated with fibrotic mechanisms in SSc. Clearly, not all patients receiving chemotherapeutic agents will develop SSc. Rather, DNA damaging agents may trigger vasculopathy and fibrosis in patients with inherent susceptibilities to SSc via a “multiple hit” mechanism (as summarized in Figure 1). This observation is not unique to chemotherapeutic agents or radiation, as other DNA damaging agents such as silica and organic solvents have been linked with SSc (37–39). Some of these risks may be present in genetic factors (40, 41) which are shared in other autoimmune diseases (42–46).

In this schema, DNA damage signals from ROS (or chemotherapeutic agents) may promote a dysregulated fibroblast phenotype characterized by increased migration and invasion. These activated fibroblasts, in turn, may promote vascular dysfunction via aberrant endothelial cell interactions (47). Similarly, aberrant DDR/R mechanisms in mesenchymal cells may promote inflammatory changes present in SSc (such as M2 macrophage polarization) (48). Indeed, taxane-based chemotherapies can result in increased levels of circulating inflammatory cytokines, such as IL-6, which are thought to be important drivers of SSc (49). DNA damage signals may also be associated with increased type I interferon production in circulating leukocytes as recently suggested by Vlachogiannis et al. particularly in patients with more progressive SSc (11). Thus, chemotherapeutic agents may potentiate fibrosis via these mechanisms in certain individuals.

### Use of HSCT in Chemotherapy Associated SSc

HSCT has been used in the treatment of autoimmune diseases unresponsive to conventional immunosuppressive therapies for decades (50). Briefly, the procedure includes chemotherapy, with or without total body irradiation, followed by the infusion of autologous (patient's own) or allogeneic (donor) stem cells intravenously to re-establish hematopoietic function in patients whose bone marrow or immune system has been damaged. These stem cells typically come from the bone marrow, peripheral blood, or umbilical cord blood (51). The mechanisms by which HSCT in SSc are unclear—although it may re-institute immune homeostasis via multiple mechanisms (52)—which perhaps may include improved inflammatory responses to DNA damage (53). In idiopathic SSc, HSCT has been shown to promote a significant improvement in skin fibrosis and mortality, in addition to a reduction of disease associated disability (54). Furthermore, HSCT improves SSc-associated vasculopathy as suggested by improved nailfold capillary loss (55). While our patient still had endorsed some shortness of breath on exertion and fatigue at ≥1 year post-transplant, these improved compared to pre-transplant. She had noticed some improvement in her skin tightening, and had decreased digital ulcerations, although her Raynaud's symptoms persisted. She, unfortunately, still had ongoing dysphagia and gastroesophageal reflux that had not improved post-transplant. Ultimately, our patient's response to HSCT was promising and brought forth the need to study the mechanism of HSCT in non-idiopathic SSc.

### Cancer, Chemotherapy and SSc in Our Patient

There has been some association of an increased risk of developing breast cancer in patients with pre-existing scleroderma (56). Cancers in SSc have been considered to stem from underlying immune dysregulation and impaired cancer immunosurveillance. In the case of our patient, symptoms began after her cancer diagnosis, there was no evidence of detectable recurrence of breast cancer, and the SSc symptoms started in the last two cycles of chemotherapy. Together, it would be less likely that her diagnosis of SSc was solely based on the underlying malignancy, although it likely contributed to it. We have also considered a paraneoplastic picture for her disease, whereby the cancer itself induced her cutaneous changes (57). However, given the timing and onset of her SSc far after her cancer diagnosis, and its onset in conjunction with her chemotherapy, paraneoplastic SSc is less likely. With all factors considered, we suspect that our patient was exposed to “multiple hits”: namely, previous history of neoplastic disease (suggesting inherently poor DDR/R mechanisms and abnormal immunosurveillance), an underlying, but poorly defined, genetic/epigenetic susceptibility for developing SSc, and finally, chemotherapeutic agents inducing DNA damage, which culminated in her development of edSSc (58).

## Conclusion

In summary, we present a case of a 39-year-old Caucasian female with chemotherapy associated edSSc, which subsequently responded to autologous HSCT. We propose that in certain individuals, particularly those with abnormal DNA repair mechanisms, such as our patient, chemotherapeutic agents may promote DNA damage signals which in turn potentiate skin fibrosis, vasculopathy, and autoimmunity. Because of the severity of her disease and how rapidly she functionally declined, she was referred for autologous HSCT, a procedure aiming to restore normal immune and mesenchymal functions resulting in a dramatic improvement. Thus, our patient reinforces the notion that HSCT may provide additional non-immunological benefits that have been previously proposed.

## Data Availability Statement

The original contributions presented in the study are included in the article/supplementary material, further inquiries can be directed to the corresponding author/s.

## Ethics Statement

Written informed consent was obtained from the individual(s) for the publication of any potentially identifiable images or data included in this article.

## Author Contributions

All authors listed have made a substantial, direct, and intellectual contribution to the work and approved it for publication.

## Funding

RG and MO's research was supported by unrestricted grants from the University of Alberta (Canada), Danish Cancer Society (Kræftens Bekæmpelse; R124-A7592 Rp12350), Canadian Dermatology Foundation (CDF), the University Hospital Foundation and Kaye Grants, and Scleroderma Canada. Additionally, MO was funded by the Arthritis Society (STAR early career development award).

## Conflict of Interest

RG receives speaker honoraria from Mallinckrodt. MO received speaker honoraria from Boehringer Ingelheim. The remaining authors declare that the research was conducted in the absence of any commercial or financial relationships that could be construed as a potential conflict of interest.

## Publisher's Note

All claims expressed in this article are solely those of the authors and do not necessarily represent those of their affiliated organizations, or those of the publisher, the editors and the reviewers. Any product that may be evaluated in this article, or claim that may be made by its manufacturer, is not guaranteed or endorsed by the publisher.
